# Effect of Si on Microstructure and Mechanical Properties of FeCrNi Medium Entropy Alloys

**DOI:** 10.3390/ma16072697

**Published:** 2023-03-28

**Authors:** Fang Ding, Yuankui Cao, Ao Fu, Jian Wang, Weidong Zhang, Jingwen Qiu, Bin Liu

**Affiliations:** 1 State Key Laboratory of Powder Metallurgy, Central South University, Changsha 410083, China; 2 College of Materials Science and Engineering, Hunan University, Changsha 410082, China; 3 Hunan Provincial Key Laboratory of High Efficiency and Precision Machining of Difficult-to-Cut Material, Hunan University of Science and Technology, Xiangtan 411201, China

**Keywords:** medium entropy alloy, microstructure, thermomechanical treatment, mechanical property, strengthening mechanism

## Abstract

FeCrNi medium entropy alloy (MEA) has been widely regarded for its excellent mechanical properties and corrosion resistance. However, insufficient strength limits its industrial application. Intermetallic particle dispersion strengthening is considered to be an effective method to improve strength, which is expected to solve this problem. In this work, microstructural evolution and mechanical behavior of FeCrNi MEA with different Si content were investigated. We found that the precipitation of fine σ particles can be formed in situ by thermomechanical treatment of Si doping FeCrNi MEAs. The FeCrNiSi_0.15_ MEA exhibits a good combination of strength and ductility, with yield strength and tensile elongation of 1050 MPa and 7.84%, respectively. The yield strength is almost five times that of the as-cast FeCrNi MEA. The strength enhancement is mainly attributed to the grain-boundary strengthening and precipitation strengthening caused by fine σ particles.

## 1. Introduction

Medium/high entropy alloys (MEAs/HEAs) are expected to be candidates for structural materials in automotive, aerospace, and vessels due to their unique microstructure and outstanding mechanical properties [1,2,3]. MEAs/HEAs with single-phase face-centered cubic (FCC) and body-centered cubic (BCC) structures are the most widely studied so far [4,5,6]. Generally, the FCC MEAs/HEAs have outstanding ductility but insufficient strength, while the BCC MEAs/HEAs show opposite mechanical properties [7,8]. Among them, the FeCrNi MEA has attracted much attention recently due to its superior combination of strength and ductility as well as corrosion resistance [9,10], which has the potential to be used as a stainless-steel material. However, the strength of the FeCrNi MEA still has great potential for further improvement.

It is well known that the introduction of secondary ceramic particles, such as carbides, borides, or silicides, is considered an effective method to enhance the strength of metallic materials [11,12]. Based on this design strategy, various interstitial elements are added to the MEAs/HEAs to induce the precipitation of the ceramic particles. For example, Chen et al. [13] reported that the incorporation of the C element during the preparation of the FeCoCrNiMn HEA by the melting method can promote the in situ formation of carbides. The tensile yield strength of the FeCoCrNiMn HEA increases significantly from 250 MPa to 650 MPa, but the plasticity drops rapidly from 52% to 12%. Xin et al. [14] found the addition of B into the Al_0.2_Co_1.5_CrFeNi_1.5_Ti_0.5_ HEA is beneficial to the precipitation of borides. The yield strength and ultimate compressive strength increase significantly, while the fracture elongation dramatically decreases to 10%. The above studies show that although the introduction of the ceramic particles can significantly enhance the strength of the MEAs/HEAs, the ceramic particles directly precipitated by the melting method are relatively coarse [15,16], which will lead to a significant deterioration in plasticity. Interestingly, recent studies found that the size, morphology, and distribution of the precipitates in the MEAs/HEAs can be further controlled through subsequent thermomechanical treatment [17,18], thus obtaining optimized strength and ductility. For instance, Liu et al. [19] found that the fine σ and μ particles can uniformly precipitate in the Mo doping FeCoCrNi HEA by cold rolling and heat treatment, which leads to a high yield strength of 816 MPa and an adequate ductility of 19%. Jo et al. [20] reported that dispersed σ particles can be formed in the V doping FeCrNi MEA through similar thermomechanical treatment conditions, and the MEA exhibits a high yield strength of 715 MPa with a retained elongation of 19.6%. Previous studies reported that Si doping in the MEAs/HEAs can improve the strength and ductility synergistically by reducing the stacking fault energy (SFE) of the matrix [21,22]. The subsequent thermomechanical treatment is expected to promote the precipitation of fine silicide to further improve the mechanical properties of the MEAs/HEAs, but relevant studies are rarely reported.

In this work, a series of the FeCrNiSi_x_ MEA (x = 0, 0.1, 0.15, 0.2) were prepared by vacuum arc melting and thermomechanical treatment. The effect of Si doping on the microstructure and mechanical properties of the FeCrNi MEA were investigated systematically.

## 2. Experimental Procedures

A series of FeCrNiSi_x_ ingots (x = 0, 0.1, 0.15, 0.2) were fabricated by vacuum arc melting from high-purity metals (>99.99%) under an argon atmosphere. All ingots were remelted five times at least to ensure uniformity. After melting, the ingots were shaped into strip samples (5 mm × 10 mm × 100 mm) by suction casting. For convenience, these ingots were named Si_0_, Si_0.1_, Si_0.15_, and Si_0.2_, respectively. Then, homogenization of the ingots was carried out at 1200 °C for 2 h and quenched with water immediately. After homogenization, the ingots were cold-rolled up to 70% thickness reduction at room temperature. Finally, the ingots were annealed at 750 °C for 1 h, and water was quenched subsequently. The achieved samples are named Si_0_-750, Si_0.1_-750, Si_0.15_-750, and Si_0.2_-750, respectively.

Phase identification was analyzed by X-ray diffraction (XRD, D/MAX-2250, Rigaku Corporation, Tokyo, Japan) with Cu Kα radiation. Microstructure was investigated by scanning electron microscopy (SEM, Tescan mira4, Tescan, Brno, Czech Republic) and transmission electron microscopy (TEM, Tecnai F20, FEI NanoPorts, Hillsboro, OR, USA) equipped with an energy dispersive spectrometer (EDS). Tensile tests were performed by an Instron 3369 machine (Instron Limited, Boston, MA, USA) with a strain rate of 1 × 10^−3^ s^−1^ at room temperature. Microhardness tests were conducted by a MicroMet-5104 (Buehler, Lake Bluff, IL, USA) machine. The applied load was 20 N and the holding time was 10 s. Each measurement was repeated three times to ensure reliability.

## 3. Results and Discussion

**Figure 1:**
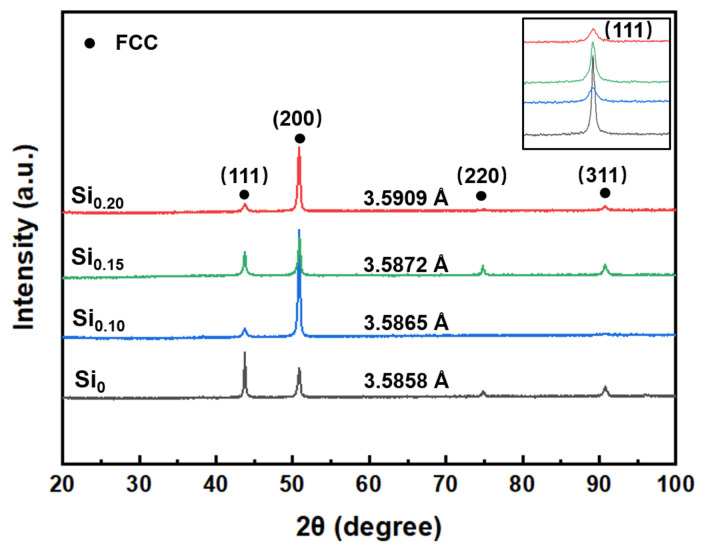
XRD patterns of the as-cast Si_x_ MEAs.

Figure 1 shows the XRD patterns of the as-cast Si_x_ MEAs. The results show that all the MEAs have a single-phase FCC structure with an increase in Si content. Meanwhile, it can be found that there is a texture in <200> direction, especially in the Si doping FeCrNi MEAs, and such casting texture is formed during the suction casting process. A similar situation has also been reported in other FCC MEAs/HEAs prepared by the casting method [19]. With a Si addition, the solidification rate slows down, resulting in a stronger casting texture. Indeed, the mechanical property is related to texture. Although the formation of <200> texture is beneficial to the improvement of the strength of the Si doping FeCrNi MEAs. However, the texture in as-cast samples is much weaker than that in as-rolled samples, which consequently causes a very limited effect on mechanical properties [23,24]. In addition, it can be seen from the inset that the diffraction peak position of (111) gradually shifts towards lower angles with an increase in Si content, implying the lattice expansion.

**Figure 2:**
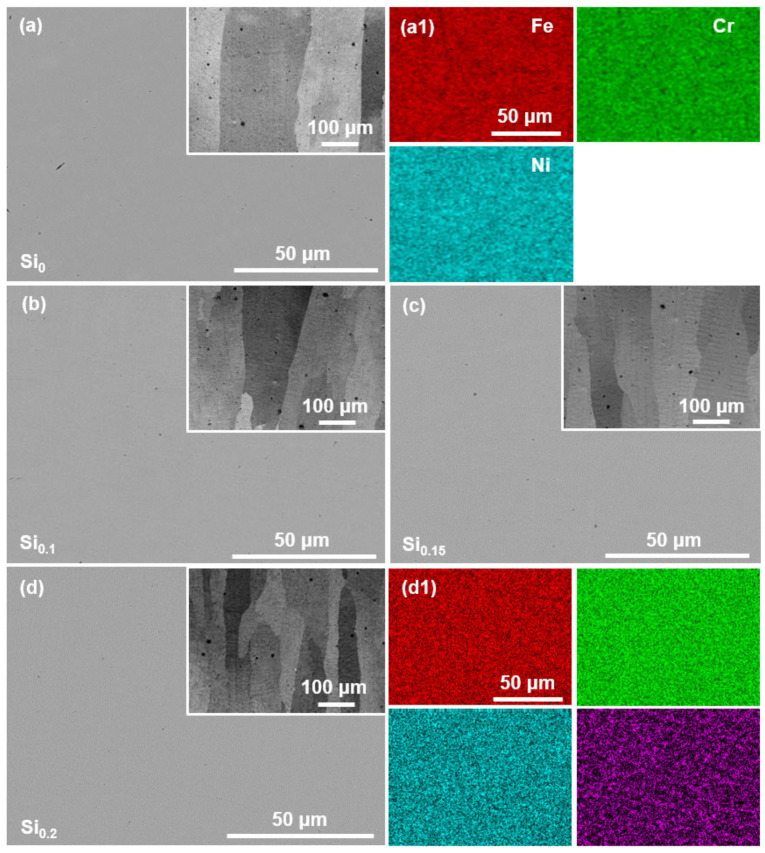
SEM images of the as-cast (**a**) Si_0_, (**b**) Si_0.1_, (**c**) Si_0.15_, and (**d**) Si_0.2_ MEAs, respectively. (**a1**) and (**d1**) show the corresponding element mapping of (**a**) and (**d**), respectively.

Figure 2 shows the SEM images and EDS elemental mapping of the as-cast Si_x_ MEAs. It can be seen that the Si_0_ MEA exhibits a single-phase microstructure, and the elements of Fe, Cr, and Ni are evenly distributed (Figure 2a,a1). Meanwhile, there is no obvious difference between the microstructure of the samples before and after Si doping (Figure 2a–d), and the secondary particles are absent in the Si doping FeCrNi MEAs. EDS elemental mapping of the Si_0.2_ MEA also indicates that the Si element is mainly dissolved in the MEA matrix rather than reacting with other elements to form silicides (Figure 2d1). Previous studies also reported that interstitial elements such as Si, C, etc., have high solid solubility in the MEAs/HEAs [11,25].

**Figure 3:**
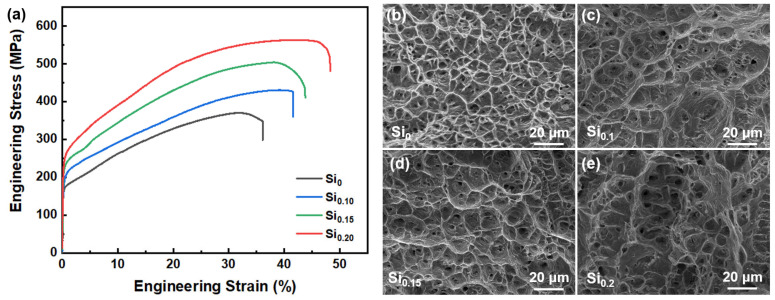
(**a**) Room-temperature tensile engineering stress-strain curves of the as-cast Si_x_ MEAs. The fracture surfaces of the as-cast (**b**) Si_0_, (**c**) Si_0.1_, (**d**) Si_0.15_, and (**e**) Si_0.2_ MEAs, respectively.

Figure 3a shows the room-temperature engineering stress-strain curves of the as-cast Si_x_ MEAs. The results show that the tensile strength and elongation of the Si_x_ MEAs increase with the increasing Si addition. It can be found the yield strength and elongation increase from 167 MPa and 36.2% of the Si_0_ MEA to 245.9 MPa and 48.4% of the Si_0.2_ MEA, respectively. This synergistic improvement of strength and ductility is mainly due to the fact that the dissolved Si atoms in the matrix can not only reduce the SFE of the MEA matrix [21,22], helping to induce more deformation mechanisms to enhance plasticity but also improve the strength of the MEA by causing a significant solid solution strengthening effect [3,25]. Figure 3b–e shows the fracture morphologies of the as-cast Si_x_ MEAs. As indicated, all the MEAs exhibit typical ductile fracture characteristics with a large number of dimples, which is attributed to the single-phase FCC structure.

**Figure 4:**
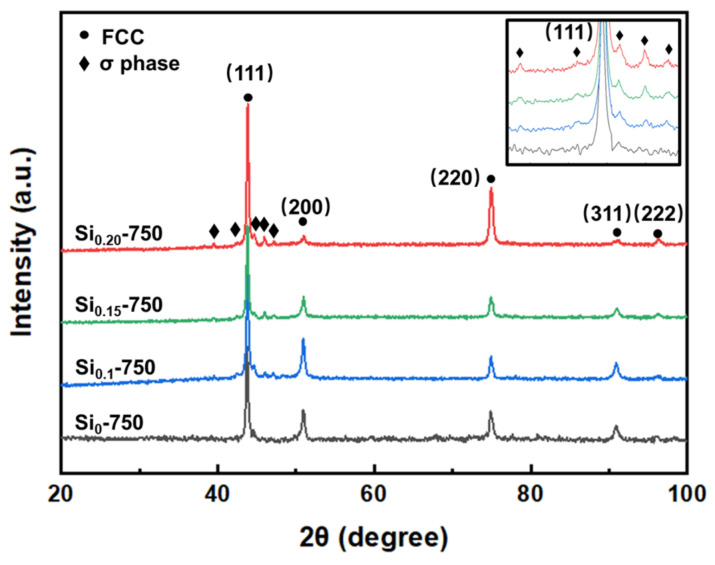
XRD patterns of the Si_0_-750, Si_0.1_-750, Si_0.15_-750, and Si_0.2_-750 MEAs.

Thermomechanical treatment was performed to promote the precipitation of fine silicides. Figure 4 shows the XRD patterns of the annealed Si_0_-750, Si_0.1_-750, Si_0.15_-750, and Si_0.2_-750 MEAs. It can be seen that the Si_0_-750 MEA is almost a single-phase FCC structure. However, additional diffraction peaks of the σ phase (JCPDS: 050708) are detected in the annealed Si doping FeCrNi MEAs. The diffraction peak intensity of the σ phase for the Si_x_-750 MEAs increases with increasing Si addition, meaning that a high Si content is beneficial to promote the precipitation of the σ phase. In addition, the texture of {111}<1$\bar{1}$0> usually forms during the rolling process, and the texture can be preserved after heat treatment. This type of texture is also observed in many rolled FCC MEAs/HEAs [26,27], which can lead to the anisotropy of mechanical properties and exhibit increased strength along the rolling direction.

**Figure 5:**
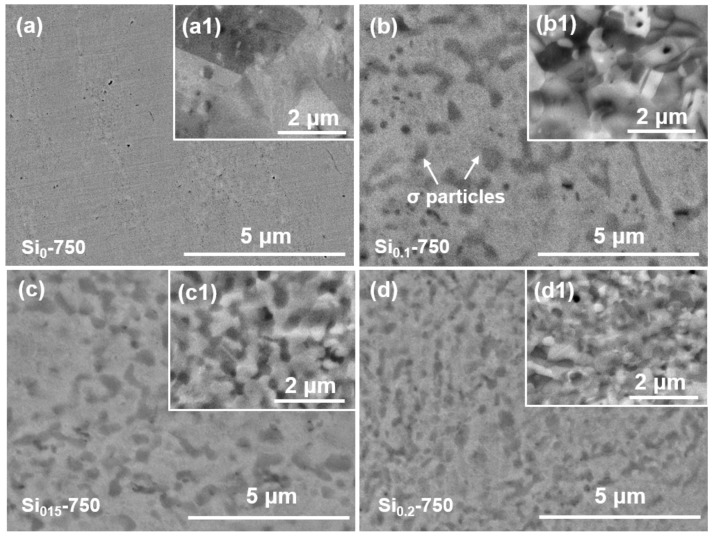
SEM images of the (**a**,**a1**) Si_0_-750, (**b**,**b1**) Si_0.1_-750, (**c**,**c1**) Si_0.15_-750, and (**d**,**d1**) Si_0.2_-750 MEAs. The inserted ECCI images show the corresponding microstructure at a high magnification view.

Figure 5a–d shows the SEM images of the Si_0_-750, Si_0.1_-750, Si_0.15_-750, and Si_0.2_-750 MEAs, respectively, and the insets show the corresponding microstructure at a high magnification view. As indicated, the Si_0_-750 MEA is mainly composed of a single-phase matrix, while the Si_0.1_-750, Si_0.15_-750, and Si_0.2_-750 MEAs consist of gray matrix and high-density fine black particles. The chemical composition in the gray and black regions is detected by EDS and listed in Table 1. It can be found that Fe, Cr, and Ni are evenly distributed in the gray matrix, while the black particle is rich in Cr and Si. According to the XRD results presented in Figure 4, the black particles are considered as the σ phase. Moreover, it can also be seen that the size and number of the σ particles are positively correlated with the amount of Si doping. The precipitation of σ particles can effectively limit the growth of grains, resulting in grain refinement [28,29]. Therefore, the grain size of the Si_x_-750 MEAs decreases with the increasing Si addition, and the Si_0.2_-750 MEA has the smallest grain size.

**Figure 6:**
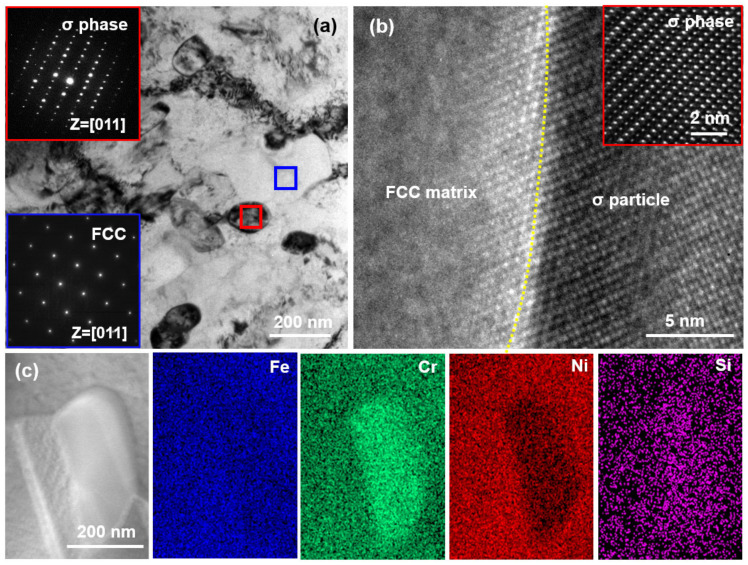
(**a**) BF image of the Si_0.15_-750 MEA. (**b**) HRTEM showing the phase interface between the FCC matrix and σ particle. (**c**) STEM EDS elemental distribution mapping showing the chemical composition of the σ particle.

The details of the microstructure for the σ particles were investigated by TEM. Figure 6a shows the bright field (BF) image of the Si_0.15_-750 MEA. Similar to the SEM results (Figure 5c), a large number of fine particles can be found in the FCC matrix (Figure 6a). The selected area electron diffraction (SAED) patterns in the red rectangle region confirm that the σ particle has a tetragonal structure [30,31]. The high-resolution transmission electron microscopy (HRTEM) of the phase interface between the FCC matrix and σ particle is presented in Figure 6b, revealing an incoherent relationship between the two phases. Moreover, the scanning transmission electron microscopy (STEM) EDS elemental mapping was performed as shown in Figure 6c to investigate the chemical composition of the σ particles, and the results show that the σ particles are (Si, Cr)-rich phase, which is consistent with previously reported results [12].

**Figure 7:**
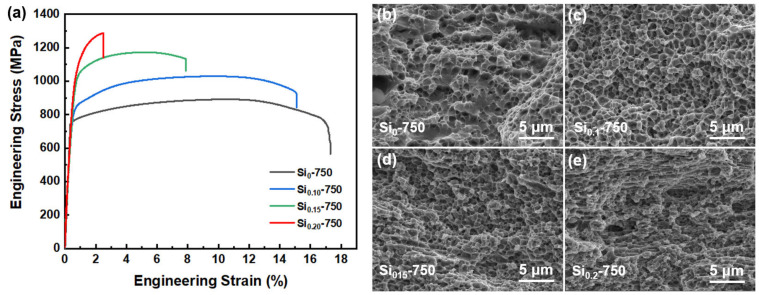
(**a**) Room-temperature tensile engineering stress-strain curves of the Si_x_-750 MEAs. The fracture surfaces of the (**b**) Si_0_-750, (**c**) Si_0.1_-750, (**d**) Si_0.15_-750, and (**e**) Si_0.2_-750 MEAs.

In order to evaluate the effect of Si on the mechanical properties of the annealed Si_x_-750 MEAs, the microhardness and tensile properties were tested at room temperature. The microhardness of the Si_0_-750, Si_0.1_-750, Si_0.15_-750, and Si_0.2_-750 MEAs are measured to be 330 HV, 352 HV, 383 HV, and 434 HV, respectively. It can be found that the microhardness of the Six-750 MEAs increase with the increasing Si addition, and the Si_0.2_-750 MEA has the maximum microhardness of 434 HV. Figure 7a shows the room-temperature engineering stress-strain curves of the Si_x_-750 MEAs. It can be seen that the yield strength (YS), ultimate tensile strength (UTS), and elongation (EL) of the Si_0_-750 MEA are 771 MPa, 1029 MPa, and 15.1%, respectively. As the Si doping increase, the strength of the Si_x_-750 MEAs increases continuously, while the elongation presents the opposite tendency. For precipitation-strengthened MEAs/HEAs, the high strength mainly comes from the grain-boundary strengthening caused by fine grains, and the precipitation strengthening results from dispersed secondary phase particles [32,33]. Compared with the other three annealed MEAs, the Si_0.2_-750 MEA has the smallest grain size and the largest σ phase number, which will lead to more significant grain-boundary strengthening and precipitation strengthening effects. Therefore, Si_0.2_-750 MEA exhibits the highest yield strength of 1088 MPa. However, the excessive addition of Si leads to the precipitation of a large number of hard but brittle σ particles along the grain boundaries of the Si_0.2_-750 MEA. The brittle σ particles distributed at the grain boundaries are prone to stress concentration and fracture during the deformation process, resulting in a significant deterioration of plasticity [34] (2.5% for the Si_0.2_-750 MEA). When the Si content drops to 0.15, the Si_0.15_-750 MEA exhibits an excellent combination of yield strength (1050 MPa) and ductility (7.84%) due to the precipitation of an appropriate amount of σ particles, and the yield strength is nearly five times that of the as-cast Si_0.15_ MEA. To further understand the strengthening mechanism of the Si_0.15_-750 MEA, the strength contribution from grain-boundary strengthening and precipitation strengthening are discussed. The strength increment induced by grain-boundary strengthening can be estimated by $\Delta \sigma_{G}{=K(d}_{{\mathrm{Si}}_{0.15}}{}^{-1/2}-d_{{\mathrm{Si}}_{0}}{}^{-1/2})$ [35], where *K* = 966 MPa·μm^−1/2^ is the Hall-Petch constant [10]; $d_{Si_{0.15}}$ = 0.9 μm and $d_{Si_{0}}\;$= 2.8 μm are the grain size of the Si_0.15_-750 MEA and Si_0_-750 MEA, respectively, which are measured from the SEM images by ImageJ software. The strength increment caused by precipitation strengthening can be estimated by $\sigma_{p}=\frac{0.81MGb}{2\pi (1-{\upsilon )}^{1/2}}\frac{ln(\pi r/4b)}{{r((2\pi /3f)}^{1/2}-\pi /2)}$ [36], where *M* = 3.06 is the Taylor factor, *G* = 79 GPa and *υ* = 0.2 are the Shear modulus and Poisson’s ratio of the FeCrNi MEA [37]; *b* = 0.254 is the Burgundy modulus; *f* = 33.5% and *r* = 150 nm are the volume fraction and average radius of the σ particles, respectively. Substituting all these parameters into the above formula, the contribution from grain-boundary strengthening and precipitation strengthening can be calculated to be 440.9 MPa and 389.8 MPa, respectively. Hence, the theoretical yield strength of the Si_0.15_-750 MEA can be estimated by $\sigma_{YS}{=\sigma }_{0}{+\Delta \sigma }_{G}{+\Delta \sigma }_{P}$ [38], where *σ*_0_ = 185.5 MPa is the lattice friction of the FeCrNi MEA [2] The theoretical yield strength can be summed to be 1016.2 MPa, which is basically equal to the experimental value (1050 MPa). The slight deviation between the theoretical value and experimental value may be attributed to the solid solution strengthening of Si in the matrix. Figure 7b–e shows the fracture morphologies of the Si_x_-750 MEAs. Consistent with the tensile results, the Si_0_-750, Si_0.1_-750, and Si_0.15_-750 MEAs all present typical ductile fracture characteristics with a large number of dimples. However, with the increase in Si content, it can be seen that the dimple size decreases and the dimple depth becomes shallower, indicating that the plasticity gradually deteriorates [39,40]. When the Si content reaches 0.2, the fracture mode changes from the initial ductile fracture to the mixed ductile-brittle fracture.

## 4. Conclusions

In this work, a series of FeCrNiSi_x_ MEAs were prepared via arc melting and thermomechanical treatment. The effect of Si on microstructure and mechanical properties was systemically studied. The main conclusions are summarized as follows:

(1) The as-cast FeCrNiSi_x_ MEAs exhibit a single-phase FCC structure. The yield strength and ductility of the FeCrNiSi_x_ MEAs increase synergistically with the increase in the Si content.

(2) Thermomechanical treatment can promote the precipitation of fine σ particles. With the increase in the Si content, the grain size of the FeCrNiSi_x_ MEAs gradually decreases, and the number of the σ particles increases significantly.

(3) As the Si doping increase, the strength of the FeCrNiSi_x_ MEAs increases continuously, while the elongation presents the opposite tendency. The FeCrNiSi_0.15_ MEA exhibits a good combination of yield strength (1050 MPa) and ductility (7.84%). The high strength is mainly attributed to the grain-boundary strengthening and precipitation-strengthening effects caused by the fine σ particles.

## Figures and Tables

**Table 1:** Chemical composition of the marked points in the Si_0.15_-750 MEA.

Regions	Fe (at%)	Cr (at%)	Ni (at%)	Si (at%)
Matrix	32.59	28.34	33.55	5.52
σ particle	24.47	47.38	12.86	15.29

## Data Availability

The data presented in this study are available on request from the corresponding author.
